# Early restoration of immune and vascular phenotypes in systemic lupus erythematosus and rheumatoid arthritis patients after B cell depletion

**DOI:** 10.1111/jcmm.14517

**Published:** 2019-07-26

**Authors:** Carlos Pérez‐Sánchez, Irene Cecchi, Nuria Barbarroja, Alejandra M. Patiño‐Trives, María Luque‐Tévar, Laura Pérez‐Sánchez, Alejandro Ibáñez‐Costa, Iván Arias de la Rosa, Rafaela Ortega, Alejandro Escudero, María Carmen Castro, Massimo Radin, Dario Roccatello, Savino Sciascia, María Ángeles Aguirre, Eduardo Collantes, Chary López‐Pedrera, BIOSAR Study Group

**Affiliations:** ^1^ Rheumatology Service, IMIBIC/Reina Sofia Hospital University of Cordoba Cordoba Spain; ^2^ Department of Clinical and Biological Sciences, and SCDU Nephrology and Dialysis, Center of Research of Immunopathology and Rare Diseases‐ Coordinating Center of Piemonte and Valle d’Aosta Network for Rare Diseases S. Giovanni Bosco Hospital Turin Italy

**Keywords:** endothelial dysfunction, inflammation, NETosis, rheumatoid arthritis, rituximab, systemic lupus erythematosus

## Abstract

This translational multi‐centre study explored early changes in serologic variables following B lymphocyte depletion by rituximab (RTX) treatment in systemic lupus erythematosus (SLE) and rheumatoid arthritis (RA) patients and investigated in vitro effects on the activity of other immune cells and the vascular endothelium. Eighty‐five SLE patients, seventy‐five RA patients and ninety healthy donors were enrolled. Two additional cohorts of selected SLE and RA patients were treated with RTX for 3 months. Changes in circulating levels of inflammatory mediators, oxidative stress markers and NETosis‐derived bioproducts were evaluated. Serum miRNomes were identified by next‐generation sequencing, and RTX‐induced changes were delineated. Mechanistic in vitro studies were performed to assess activity profiles. Altered inflammatory, oxidative and NETosis‐derived biomolecules were found in SLE and RA patients, closely interconnected and associated to specific miRNA profiles. RTX treatment reduced SLE and RA patients' disease activity, linked to a prominent alteration in those biomolecules and the reversal of altered regulating miRNAs. In vitro studies showed inhibition of NETosis and decline of pro‐inflammatory profiles of leucocytes and human umbilical vein endothelial cells (HUVECs) after B cell depletion. This study provides evidence supporting an early RTX‐induced re‐setting of the pro‐inflammatory status in SLE and RA, involving a re‐establishment of the homeostatic equilibrium in immune system and the vascular wall.

## 1 INTRODUCTION

The presence of autoantibodies, and therefore the role of B cells, is a fundamental characteristic of both rheumatoid arthritis (RA) and systemic lupus erythematosus (SLE).^1^, ^2^ One of the main traits of both diseases, apart from a “vicious cycle” of inflammation, is determined by the interaction between B‐ and T cells.^1^ In fact, in addition to antibody‐dependent mechanisms, B cells act as antigen‐presenting cells and co‐stimulate T lymphocytes and other immune cells, including monocytes and neutrophils. This ultimately results in the increase of oxidative stress, neutrophils extracellular traps activation and release (NETosis), and the delivery of cytokines and cell‐surface ligands, which in turn provides “help” to other B cells.^3^, ^4^, ^5^ Recent studies have investigated the epigenetic processes implicated in inflammation, including an altered expression of microRNAs (miRNAs) that might play a key role in regulating inflammation.^6^, ^7^, ^8^

Based on the evidence supporting the crucial role of B cells in the pathophysiology of these two autoimmune diseases,^1^, ^2^ many investigations have evaluated the clinical impact of B cell–targeted therapies. Rituximab (RTX) is a monoclonal antibody directed at CD20 molecules expressed on the surfaces of pre‐B and mature B lymphocytes. The mechanisms of RTX action include the following: cytolysis, through complement or antibody‐dependent cell‐mediated cytotoxicity, induction of apoptosis, blockade of co‐stimulatory molecules, and neutralization of pathogenic antibodies and soluble factors such as cytokines and their receptors.^9^

While the role of RTX in controlling the clinical manifestations and disease progression of RA and SLE has been widely documented^9^ to date, the exact mechanisms underlying the complex interplay between B cell depletion and pro‐inflammatory status leading to clinical response need further elucidation.

In this study, we aimed to explore changes in serologic parameters related to inflammation, oxidative stress, NETosis and regulating miRNAs, following B lymphocyte depletion in RA and SLE patients, along with their effects on the activity of other immune cells and the vascular endothelium.

## 2 MATERIALS AND METHODS

### 2.1 Patients

Seventy‐five RA patients, eighty‐five SLE patients and ninety healthy donors (HD) were included in the study (during a 24‐month period). Additionally, two independent cohorts of sixteen consecutive RA patients and sixteen SLE patients treated with RTX were studied. Both cohorts attended the Reina Sofia University Hospital of Cordoba (Spain) and the St. Giovanni Bosco Hospital of Turin (Italy). All patients fulfilled the American College of Rheumatology revised criteria for SLE^6^ or RA.^7^ Approval from the ethics committees was obtained, and subjects provided written informed consent. Clinical/laboratory parameters of patients are displayed in Table S1. Clinical/laboratory profiles of the patients treated with RTX are displayed in Table 1.

**Table 1:** Clinical and laboratory profiles of RA and SLE patients before and after RTX treatment

	Healthy donors (n = 26)	SLE before RTX (n = 16)	*P* *	SLE after RTX (n = 16)	*P* ^#^	RA before RTX (n = 16)	*P* *	RA after RTX (n = 16)	*P* ^#^
Demographic characteristics									
Female/male (n)	17/8	14/2				10/6			
Disease evolution (y, mean ± SD)		9 ± 8				16 ± 12			
Age (y, mean ± SD)	43 ± 14	45 ± 10	n.s			52 ± 16	n.s		
Clinical characteristics									
Smoking (n, %)	6 (23.1%)	4 (25%)	n.s			5 (31.2%)	n.s		
Hyperlipidemia (n, %)	4 (15.3%)	6 (37%)	**0.01**			3 (18.7%)	n.s		
Arterial hypertension (n, %)	3 (11.5%)	7 (43%)	**0.01**			2 (12.5%)	n.s		
Nephropathy (n, %)	0/26 (0%)	13/16 (81.2%)	**0.00**			1/16 (6.2%)	n.s		
Autoimmune profile									
ACPAs, IU/mL (mean ± SD)	4.5 ± 6.5					632.9 ± 730	**0.01**	359.2 ± 565	n.s
RF, IU/mL (mean ± SD)	1.5 ± 2.8					385.1 ± 317.7	**0.01**	207.8 ± 130	**0.04**
Anti‐dsDNA (n, %)	0 (0%)	13 (81.2%)	**0.00**	2 (12.5%)	**0.00**				
Antiphospholipid antibodies (n, %)	0 (0%)	1 (6.2%)	n.s	1 (6.2%)	n.s				
Laboratory parameters									
CRP (mg/L) (mean ± SD)	1.6 ± 1.6	8.2 ± 8.6	**0.01**	0.5 ± 0.3	**0.01**	12.6 ± 10.4	**0.00**	3.2 ± 1.6	**0.03**
ESR (mm/h) (mean ± SD)	7.6 ± 5.8	59 ± 14	**0.00**	20 ± 14	**0.00**	24.3 ± 15	**0.00**	11.3 ± 7.6	**0.04**
C3 (mg/dL) (mean ± SD)	125 ± 45	58.8 ± 28	**0.00**	98.1 ± 22.3	**0.00**	137.5 ± 30.1	n.s	116.4 ± 18	n.s
C4 (mg/dL) (mean ± SD)	31.6 ± 25.2	9.4 ± 8.2	**0.00**	20.2 ± 7.1	**0.00**	29.8 ± 14.6	n.s	17.06 ± 5	n.s
Disease activity assessment									
DAS28 (mean ± SD)						4.8 ± 1		3.2 ± 0.9	**0.04**
SLEDAI‐2K (mean ± SD)		8.5 ± 3		1.5 ± 2	**0.00**				
Treatments									
NSAIDs (%)						15/16 (93.7%)			
Corticosteroids (n, %)		15 (93.7%)				14 (87.5%)			
Statins (n, %)		1 (6.2%)				2 (12.5%)			
Immunosuppressors (n, %)		9 (56.2%)				15 (93.7%)			
Hydroxychloroquine (n, %)		11 (68.7%)							
Anticoagulants/antiplatelets agents (n, %)		4 (25%)							

Bold values identify statistical significance. Abbreviations: ACPAs, anti‐citrullinated protein antibodies; CRP, C‐reactive protein; DAS28, RA Disease Activity Score; ESR, erythrocyte sedimentation rate; NSAIDS, non‐steroid anti‐inflammatory drugs; RA, rheumatoid arthritis; RF, rheumatoid factor; SLE, systemic lupus erythematosus; SLEDAI‐2K, Systemic Lupus Erythematosus Disease Activity Index.

* Indicates significant differences vs healthy donors.

^#^ Indicates significant differences vs patients before RTX (*P* < 0.05).

Patients were treated with RTX as rescue therapy because they were intolerant/refractory to standard immunosuppressants. The therapy with RTX was based on treating physicians’ judgement and agreed case‐by‐case by multidisciplinary evaluation. RTX regimens included two infusions of RTX 1 g 2 weeks apart (in RA patients), or 375 mg/m^2^ weekly for 4 weeks (in SLE patients).

### 2.2 Blood collection and assessment of inflammatory, oxidative stress and NETosis parameters

Plasma and serum samples and purified leucocytes were obtained as previously reported.^10^, ^11^ Inflammatory mediators, oxidative stress parameters and NETosis were analysed as previously described.^8^, ^12^ For further details, see Appendix S1.

### 2.3 RNA and miRNA isolation, profiling and quantitative real‐time PCR

Total RNA from lymphocytes, monocytes, neutrophils and human umbilical vein endothelial cells (HUVECs) was extracted using TRI Reagent (Sigma). Inflammatory genes were evaluated as described elsewhere.^10^ List of primers used is displayed in Table S2. Total RNA, including the miRNA fraction, was extracted from serum by using the QIAzol miRNeasy kit (Qiagen), as previously reported.^13^ The protocols and primers used for both, miRNA profiling and RT‐PCR, are described in the Appendix S1.

### 2.4 In vitro study with RTX on lymphocyte population

Dose‐ and time‐response experiments were carried out on lymphocytes purified from RA patients**.** Two doses of RTX (1 and 10 μg/mL) at 2× (24 and 48 hours) were evaluated in parallel, based on previous studies.^14^, ^15^, ^16^

Lymphocytes purified from eight SLE and eight RA patients were incubated in vitro with 1 µg/mL of RTX for 24 hour. SLE and RA patients selected had high activity scores (mean SLEDAI‐2K of eight and mean DAS28 of 4.7, respectively).

The percentages of B cells before and after RTX treatment were analysed by flow cytometry on a flow cytometer FACSCalibur after incubation with PE human anti‐CD19 and FITC human anti‐CD3 (Miltenyi Biotech) as previously described.^17^ Changes in gene expression of selected pro‐inflammatory molecules (IL‐1β, IL‐6, IFN‐γ and TNF‐α) were evaluated using RT‐PCR.

### 2.5 In vitro treatment of HUVECs, monocytes and neutrophils from HDs with serum from SLE and RA patients before and after 3 months of RTX therapy

Human umbilical vein endothelial cells (HUVECs) were cultured in Endothelial Cell Basal Medium (EBM; Lonza) with 10% FBS, 0.1% human epidermal growth factor (hEGF), 0.1% hydrocortisone, 0.1% gentamicin, amphotericin‐B (GA‐1000), 0.4% bovine brain extract, 100 U/mL penicillin, 100 mg/mL streptomycin and 250 pg/L fungizone (BioWhittaker) at 37°C in a humidified 5% CO_2_ atmosphere.

Monocytes and HUVECs were incubated for 24 hours with serum (10%) obtained from SLE and RA patients at baseline and after 3‐month RTX therapy; neutrophils were treated only for 6 hours to avoid the effect of the naturally occurring apoptotic process.

### 2.6 Statistical analysis

All data were expressed as mean ± SD or median [range] according to their distribution. Following normality and equality of variance tests, clinical characteristics were compared using paired Student's *t* test or alternatively by a non‐parametric test (Mann‐Whitney rank sum test). Paired samples within the same subjects were compared by Wilcoxon signed‐rank test. Correlations were assessed by Spearman's rank. Statistical analyses were performed with SPSS 19.0 (SPSS Inc).

## 3 RESULTS

### 3.1 Early clinical response to RTX therapy

Clinical and analytical assessments after 3 months of RTX therapy revealed that 81.25% of SLE patients were early responders to the treatment (mean SLEDAI‐2K after treatment = 1.5). The mean levels of acute‐phase reactants (CRP and ESR), complement factors C3 and C4, and anti‐dsDNA levels were also significantly reversed (Table 1).

Among RA patients, 62.5% showed early positive response to RTX therapy, as demonstrated by the reduction of disease activity (mean DAS28 of 3.2). Mean levels of RF and acute‐phase reactants were also significantly reduced (Table 1).

### 3.2 Serum inflammatory, oxidative stress and NETosis‐derived parameters are deregulated, closely linked and associated with the clinical and autoimmune profiles of SLE and RA patients

**Figure 1:**
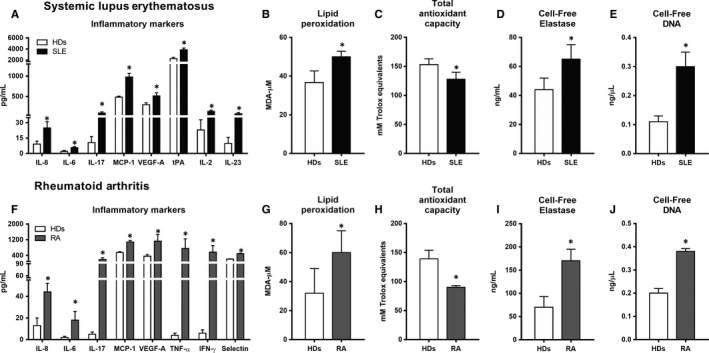
Serum inflammatory, oxidative stress and NETosis markers in SLE and RA patients. Cytokines/inflammatory markers in systemic lupus erythematosus (SLE, A) and rheumatoid arthritis (RA, F). Lipid peroxidation in SLE (B) and RA (G). Total antioxidant capacity in SLE (C) and RA (H). Neutrophil‐derived elastase in SLE (D) and RA (I). Cell‐free DNA in SLE (E) and RA (J). Bar graphs represent mean ± SD; parameters were compared to healthy donors (HDs). (*P < 0.05). ICAM, intercellular adhesion molecule; IFN‐γ, interferon gamma; IL, interleukin; MCP‐1, monocyte chemotactic protein‐1; TNF‐α, tumour necrosis factor alpha; tPA, tissue plasminogen activator; VEGF‐A, vascular endothelial growth factor A

Patients with SLE showed increased serum levels of VEGF, IL‐2, IL‐6, IL‐8, IL‐17, IL‐23, MCP‐1 and tPA compared with HD (Figure 1A). Patients with SLE also displayed a pro‐oxidative status, and LPO levels were found to have increased (Figure 1B), indicative of an abnormally elevated reaction of peroxides with lipids in plasma. TAC was found to be reduced in plasma vs HD, indicating a reduced ability to counteract ROS and resist oxidative damage (Figure 1C).

An inflammatory and oxidative status was also demonstrated in the serum of RA patients, including over‐expression of IL‐6, IL‐8, IL‐17, MCP‐1, TNF‐α, IFN‐γ, sP‐selectin and VEGF (Figure 1F), increased levels of LPO (Figure 1G) and reduced TAC (Figure 1I).

Increased NETs extrusion was demonstrated by enlarged neutrophil cell‐free elastase and cell‐free DNA plasma levels in both SLE and RA patients (Figure 1D‐E and 1‐J, respectively). Moreover, a significant positive correlation was demonstrated among both parameters in the two diseases (data not shown).

Correlation studies demonstrated a strong relationship among the levels of all the parameters evaluated, including inflammatory and oxidative stress markers, as well as with NETosis‐derived products (data not shown).

**Figure 2:**
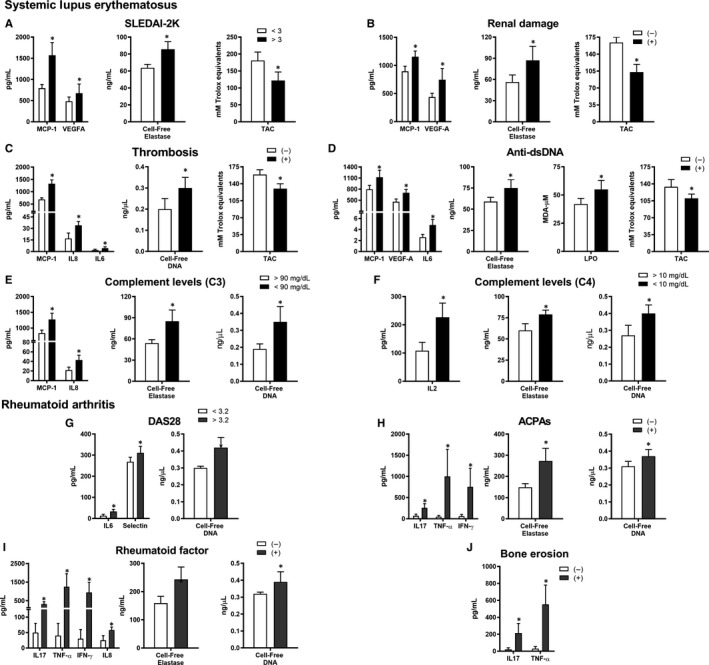
Association studies in SLE and RA patients. Association of the altered inflammation, oxidative stress and NETosis markers with systemic lupus erythematosus (SLE) disease activity (SLEDAI‐2K) (A), renal damage (B), thrombosis (C), positivity for anti‐dsDNA antibodies (D) and hypocomplementemia C3/C4 (E‐F). Association of the altered inflammation, oxidative stress and NETosis markers with the RA disease activity (DAS28 > 5) (G), positivity for anti‐citrullinated protein antibodies (ACPAs) (H), positivity for rheumatoid factor (I) and bone erosion (J). Bar graphs represent the mean ± Standard Deviation. (*P < 0.05)

NETosis‐derived products, such as cell‐free elastase and cell‐free DNA levels, were strongly associated with clinical parameters in SLE patients, including high SLEDAI‐2K (Figure 2A), renal damage (Figure 2B), thrombosis (Figure 2C) and hypocomplementemia (Figure 2E‐F). Positivity for anti‐dsDNA antibodies was associated with plasma inflammation markers, as well as with bioproducts of oxidative stress (Figure 2D). A direct relationship with the levels of cell‐free elastase was further verified (Figure 2D).

In RA patients, a direct relationship among high disease activity (DAS28 > 3.2) and some inflammatory parameters, as well as with cell‐free DNA levels, was demonstrated (Figure 2G). Accordingly, positivity for ACPAs was associated with plasma levels of IL‐17, TNF‐α and IFN‐γ (Figure 2H).

Significant associations were found among the RA autoimmune profile and the levels of both cell‐free elastase and cell‐free DNA, including the positivity for ACPAs antibodies (Figure 2H) and RF (Figure 2I). Moreover, associations among the presence of bone erosion and higher levels of inflammatory molecules (IL‐17 and TNF‐α) were also confirmed (Figure 2J).

### 3.3 Changes in the inflammatory parameters, oxidative stress and NETosis in the serum of SLE and RA patients after RTX therapy

**Figure 3:**
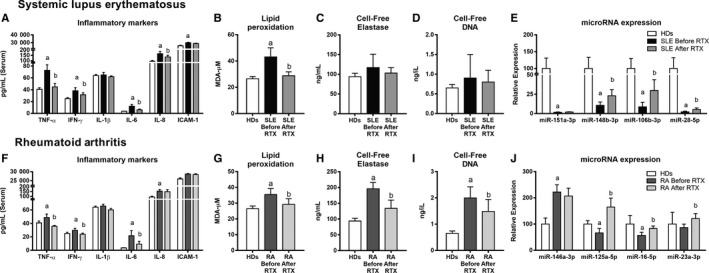
Inflammation, oxidative stress, NETosis and miRNAs in systemic lupus erythematosus (SLE) and rheumatoid arthritis (RA) patients after rituximab (RTX) therapy. All measures were performed in patients’ serum before and after RTX treatment. Bar graphs represent the mean ± SD of inflammatory molecules in HDs, SLE (A) and RA patients (E); lipoperoxides (LPO) in SLE (B) and RA (F); cell‐free elastase in SLE (C) and RA (G); cell‐free DNA levels in SLE (D) and RA (H). miRNAs expression in SLE (E) and RA (J). (a) significant differences vs HDs (*P* < 0.05). (b) significant differences vs before RTX (*P* < 0.05)

After 3 months of RTX treatment, the levels of pro‐inflammatory molecules were significantly reduced (Figure 3A and 3). This is in line with the observed reduction of disease activities in both SLE and RA patients (Table 1). 85% of RA patients and 90% of SLE patients were positive responders to therapy. Therefore, no comparative molecular analyses among responders and non‐responders could be performed.

Levels of RF, anti‐dsDNA and acute‐phase reactants were also reduced. RTX further reduced the serum levels of LPO (Figure 3B and G). NETosis‐derived products showed a trend of reduction in SLE patients (Figure 3C‐D) and reached statistical significance in RA (Figure 3H‐I).

### 3.4 Modulation of altered circulating miRNAs in SLE and RA patients after RTX therapy

The analysis of the whole miRNome profile of SLE patients after RTX treatment showed altered serum expression of 71 miRNAs (cut‐off: 2‐fold change), including 42 up‐regulated and 29 down‐regulated (Figure S1A). By using bioinformatics tools, we identified a set of miRNAs that might modulate the expression of relevant genes related to clinical features of SLE patients. Thus, a network that integrates the interaction of miRNA‐mRNA target was performed (Figure S2A). The miRNAs selected (miR‐151a‐3p, miR‐148b‐3p, miR‐106b‐3p and miR‐28‐5p) were validated as reduced in the serum of all the recruited SLE patients. Furthermore, after RTX therapy, their levels were significantly up‐regulated (Figure 3E).

In the miRNome of RA, the serum expression levels of 198 miRNAs were found significantly altered (cut‐off: 2‐fold change) in the exploratory phase, including 114 up‐regulated and 84 down‐regulated (Figure S1B). The in silico study allowed to select four miRNAs, including miR‐146a‐3p, miR‐125a‐5p, miR‐16‐5p and miR‐23a‐3p, as potential modulators of mRNA targets involved in the pathogenesis of RA (Figure S2B). Besides, the increased levels of miR‐146a‐3p and the reduced levels of miR‐125a‐5p, miR‐16‐5p and miR‐23a‐3p found in the array were validated by RT‐PCR in the whole cohort of RA patients. After RTX therapy, the levels of those miRNAs were significantly reversed (Figure 3J).

### 3.5 Correlation and association studies

In the cohorts of SLE and RA patients treated with RTX, association studies showed that the basal levels of deregulated circulating parameters related to inflammation, oxidative stress and NETosis were interrelated and linked to a number of clinical features (Figure S3).

Circulating levels of microRNAs validated in SLE patients were found linked to levels of inflammatory parameters, as well as to clinical features, including high activity of the disease or hypocomplementemia (Figure S4A‐B).

In RA patients, similar profiles of correlation were demonstrated, involving a direct relationship between levels of the circulating miRNAs validated, inflammatory molecules and NETosis‐derived products, as well as with clinical and autoimmune parameters such as activity of the disease and positivity for RF and ACPAs (Figure S4C‐D).

Accordingly, the changes in levels of specific microRNAs after RTX treatment on each autoimmune disorder paralleled the changes in several inflammatory molecules and NETosis‐derived products, along with the normalization of clinical features (ie reduction of autoantibodies, improvement in the activity of the disease or reversed hypocomplementemia, among others; Table S4).

### 3.6 In vitro effects of RTX on the inflammatory profile of lymphocytes from HDs, SLE and RA patients

Time‐ and dose‐response studies on lymphocytes from RA patients treated with RTX showed the effectiveness of the treatment through reduction of B lymphocytes (CD19+) and the inflammatory status of the lymphocyte population (Figure S5). Thus, the lowest dose (1 µg/mL) and the shortest time (24 hours) were selected to perform all the in vitro studies.

**Figure 4:**
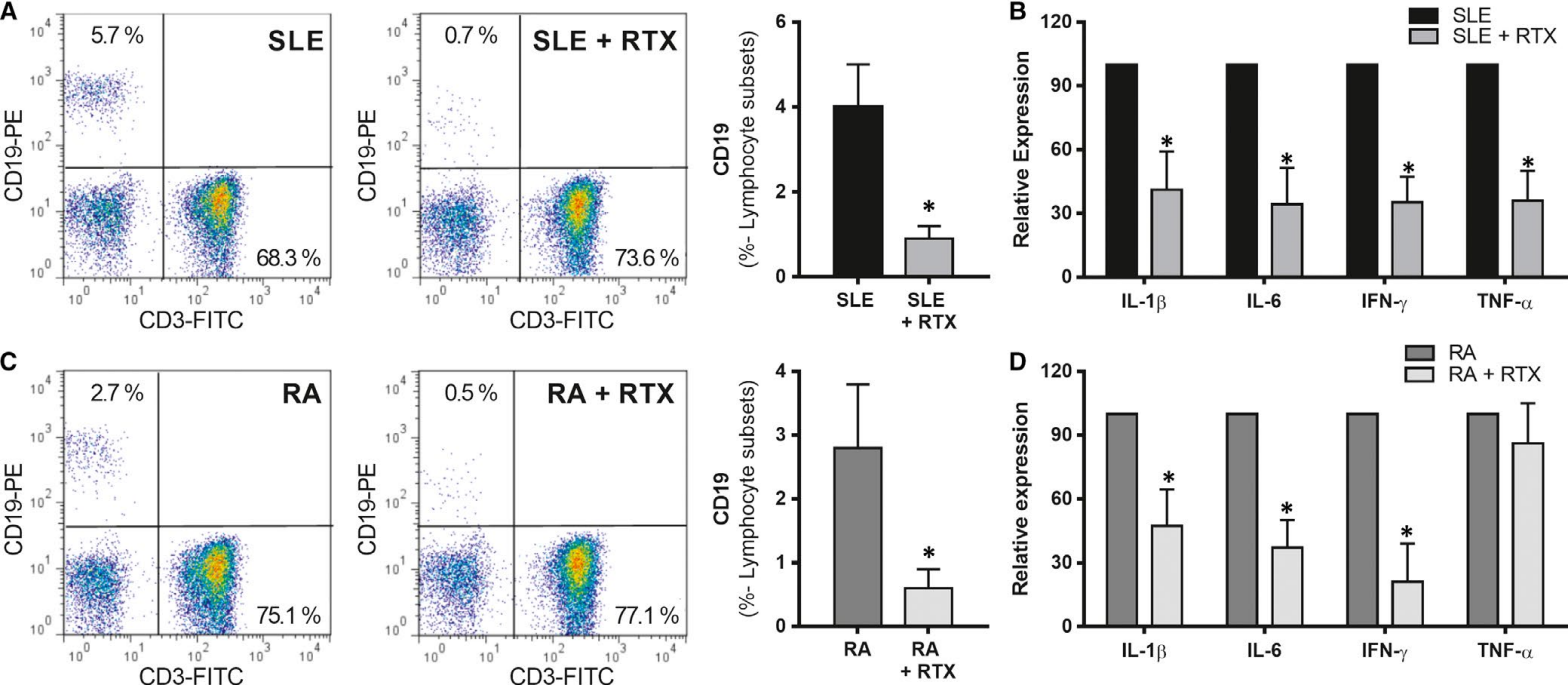
In vitro effects of rituximab (RTX) on lymphocyte population. Flow cytometry dot plot (CD19‐PE/CD3‐FITC) representative of B cell depletion by effect of RTX treatment (1 µg/mL for 24 hours) on lymphocyte populations from 8 SLE (A) and 8 RA patients (C). The pro‐inflammatory gene profiles of lymphocytes from SLE (B) and RA patients (D) were found reduced after RTX in vitro treatment. Bar graphs represent mean ± SD. (*P < 0.05)

The in vitro treatment of SLE and RA purified lymphocytes with 1 µg/mL of RTX for 24 hours and reduced the percentage of B lymphocytes (Figure 4A and 4). The RTX treatment was also effective in reducing the levels of a cytokine panel integrated by IL‐1β, IL‐6, IFN‐γ and TNF‐α on the lymphocyte population from SLE and RA patients (Figure 4B and 4).

### 3.7 In vitro changes in the activation state of endothelial cells and monocytes induced by serum from SLE and RA patients treated with RTX

The in vitro treatment of monocytes with SLE and RA serum before RTX treatment promoted increased expression of pro‐thrombotic and pro‐inflammatory molecules (TF, VEGF, MCP‐1, IL‐8, IL‐1ß). Besides, the treatment with the serum of those patients after RTX therapy abridged this activation status on healthy monocytes (Figure S6A‐B).

Similarly, the in vitro treatment of HUVECs with serum from SLE and RA patients before RTX therapy promoted the increased expression of inflammatory mediators such as VEGF, IL‐8, ICAM‐1 and endothelial nitric oxide synthase. The incubation with SLE and RA serum after RTX therapy significantly reduced the expression of the markers with HUVECs’ activation (Figure S6C‐D).

### 3.8 In vitro effects of serum from SLE and RA patients treated with RTX on the induction of NETosis and the pro‐inflammatory state of neutrophils

**Figure 5:**
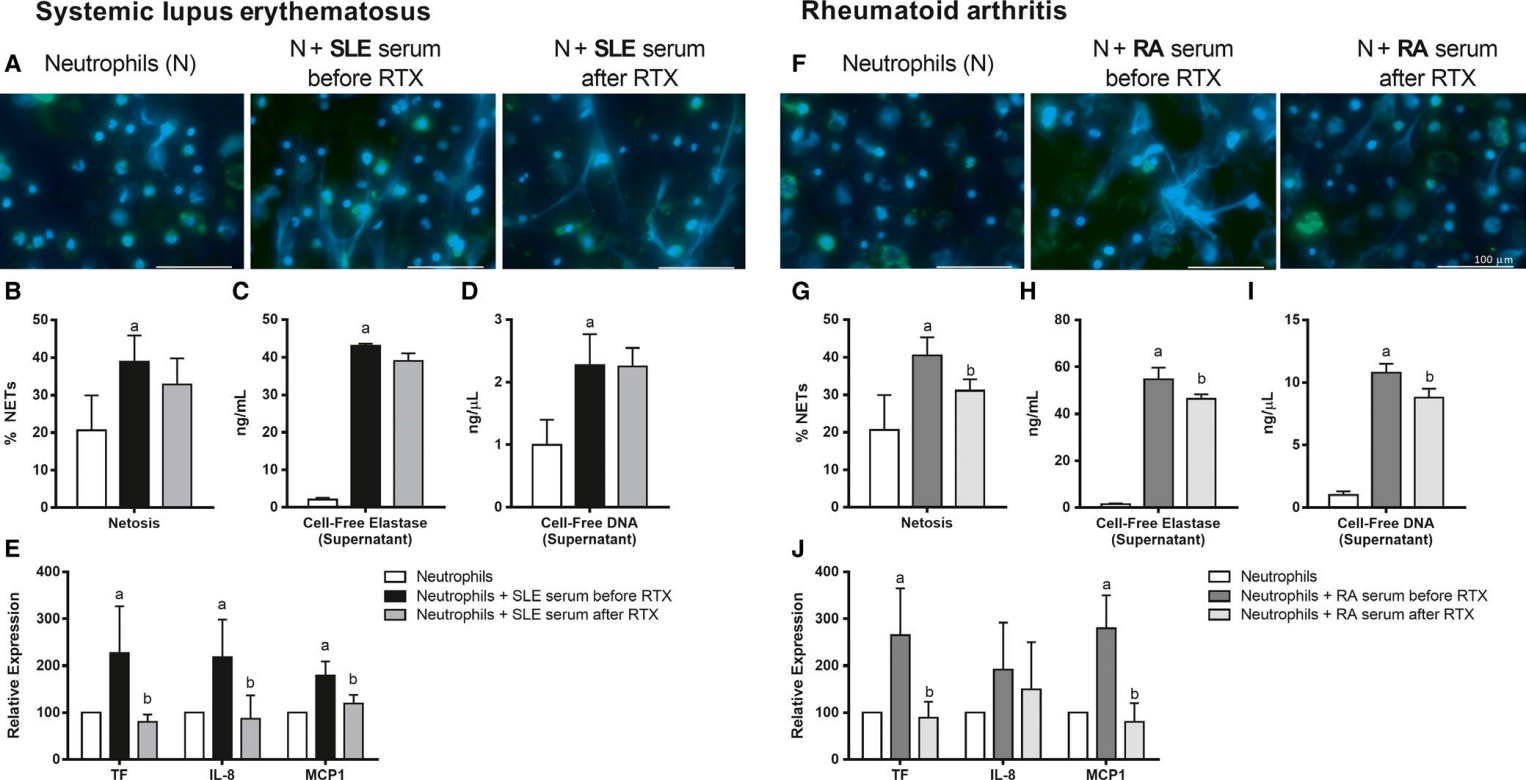
Modulation of NETosis and pro‐inflammatory profile of neutrophils by RTX. HD neutrophils treated with systemic lupus erythematosus (SLE, A) and RA patients' (F) sera before and after RTX therapy. Elastase and DAPI are shown in green and blue, respectively. Scale bar 100 μm (A‐F). Results were expressed as NETs(%) ± SEM of 20 randomly selected fields (B‐G). Cell‐free elastase and DNA in supernatant of neutrophils treated with serum from SLE (C‐D) and RA patients after RTX therapy (H‐I). Inflammatory markers of serum‐treated healthy neutrophils (E‐J). Bar graphs represent the mean ± SD. (a) significant differences vs untreated neutrophils (*P* < 0.05). (b) significant differences vs serum before rituximab (RTX) treatment (*P* < 0.05)

Neutrophils from HDs, cultured in the presence of RA and SLE serum, showed a significant induction of NETosis, as well as in the levels of NETosis‐derived products released to the cell culture medium, including elastase and DNA. The NETotic process and the derived products released were reduced when the neutrophils were treated with SLE and RA serum after RTX therapy (Figure 5A‐D and F‐I).

In parallel, the serum from RA and SLE patients before RTX therapy promoted a pro‐inflammatory status in neutrophils, demonstrated by the increased expression of TF, IL‐8 and MCP‐1. The incubation with SLE and RA serum after RTX therapy mitigated this activation process (Figure 5E and J).

## 4 DISCUSSION

In the present study, we demonstrated that RTX induced an early re‐setting of the immune and vascular system status involving a significant re‐establishment in the levels of circulating inflammatory and oxidative stress mediators, as well as a substantial reduction of NETosis‐derived products and a prominent reversal of altered miRNAs regulating those molecules. Similar profiles and responses were found in both disorders, from which clinical features most probably depend on specific autoantibodies.

It has been reported that RTX administration may decrease the activated phenotype of peripheral and tissue‐resident T cells by abolishing antigen presentation by B cells and may enhance the numbers and function of regulatory T cells (Treg).^18^ Moreover, it has been shown that after B cell depletion, the remaining and/or regenerating B cells develop an incompletely understood deficiency in co‐stimulatory molecule expression, enhancing the lack of co‐stimulation provided to autoantigen‐specific T cells.^18^ Accordingly, we observed that RTX reduced serum pro‐inflammatory cytokines produced by immune cells, such as T lymphocytes, including TNF‐α, IFN‐γ and IL‐6. These cytokines have been demonstrated to play key roles in the pathophysiology of both diseases, so that their elevated serum levels in RA patients correlated with radiological progression and disease activity,^19^ while in SLE patients they influence disease activity, arthritis and neuropsychiatric manifestations.^20^, ^21^ Results were further supported by our in vitro assays, showing that lymphocytes from SLE and RA patients treated with RTX displayed a significantly reduced expression of those inflammatory mediators.

Monocytes, central players in inflammation and thrombosis, have been found to be activated in SLE and RA through the release of cytokines, chemokines and pro‐thrombotic factors, and massive infiltration in inflammatory sites, such as synovial membranes, kidneys and atherosclerotic plaques.^10^, ^22^ Accordingly, in vitro incubation of HDs monocytes with serum from active RA or SLE patients induced increased expression of pro‐thrombotic and pro‐inflammatory molecules. Interestingly, such changes were not observed after incubation with serum from patients treated with RTX. It is likely that, in those patients, the B cell depletion and the resulting reduced secretion of pro‐inflammatory mediators by immune cells (ie IL‐6, IFN‐γ, TNF‐α and IL‐8) might decrease the levels of these monocyte‐activating molecules 12, 23 in the serum of treated SLE and RA patients, thus preventing the activation of this cell population. A similar effect might be also mediated by a reduced production of NETosis bioproducts (ie cell‐free elastase and cell‐free DNA), lipid peroxidation, as well as the demonstrated increase in number and function of Treg cells, which further inhibit T cell proliferation and cytokine production.^18^

Previous studies from our group have proved the existence of an altered oxidative status in plasma and leucocytes from antiphospholipid syndrome, SLE and RA patients, further reversed by in vivo ubiquinol, statins and tocilizumab treatments, respectively.^11^, ^22^, ^24^ In the present study, the reduced pro‐oxidative status after RTX therapy most likely arises from the parallel inhibition of the autoantibodies and pro‐inflammatory molecules production, as demonstrated by the decrease of basal levels of oxidative products and antioxidant activity.

Rheumatoid arthritis ‐ and SLE‐derived neutrophils are prone to undergo NETosis. NETs themselves could contribute to the generation of autoantigens, or serve as autoantibodies targets, leading to a self‐perpetuating mechanism of disease in these autoimmune conditions.^25^, ^26^ Accordingly, a recent study by our group demonstrated that elements associated with the extrusion of NETs are significantly enhanced in RA patients and that NETosis‐derived products correlated with clinical, inflammatory and oxidative stress markers. Previous experiences showed that biological therapies directly targeting IL‐6 receptor and TNF‐α have a significant effect on NETosis inhibition, which paralleled the reduction of disease activity and inflammatory mediators.^12^ Hence, we demonstrated a remarkable effect of B cell depletion on NETosis prevention, particularly in RA patients. In vitro studies further demonstrated a significant inhibition of NETs extrusion, along with the reduced expression of inflammatory mediators by these cells.

Our results are also in accordance with a very recent study by Kraaij and colleagues,^27^ which demonstrated that therapeutic intervention in SLE patients with a combination of RTX and belimumab effectively reduced anti‐nuclear antibodies (ANAs) and regressed excessive NET formation ex vivo, while achieving significant clinical responses in patients with severe refractory SLE.

It has been demonstrated that NETosis in RA and SLE consist on a “vicious cycle” on which NETs are generated through stimulation of neutrophils by autoantibodies and inflammatory mediators and, in turn, generate new autoantigens for autoantibody production, leading to a self‐perpetuating mechanism of disease in these autoimmune disorders.^28^

Furthermore, it is likely that the depletion of B cells in SLE and RA patients drives the reduction of NETosis, which, in turn, contributes to the reduction of inflammatory mediators and autoantibody production, leading to the improvement of the disease.

Overall, at the molecular level, the underlying effects of RTX might involve a concomitant and interrelated reduction of NETosis, along with autoimmune, inflammatory and oxidative stress mediators. This hypothesis was supported by the direct relationship found in vivo and in vitro between the reduction of NETs extrusion products in serum from RA and SLE patients and the decline of disease activity, levels of autoantibodies, acute‐phase reactants, pro‐thrombotic and pro‐inflammatory mediators and bioproducts of oxidative stress.

B cell depletion in RA setting leads to ameliorated endothelial function, along with a decrease of disease activity,^29^, ^30^ carotid intima media thickness and atherosclerosis progression.^31^ Moreover, RTX therapy might have positive effects on subclinical atherosclerosis, as early as at 6 months, associated to the reduction of IgM RF, an independent predictor of cardiovascular mortality.^32^ In relation to previous reports, our study further delineates the influence of RTX on the regulation of the vascular system in the context of SLE and RA. This is realized by promoting in vitro the inhibition of molecules related to inflammation, angiogenesis and cell adhesion in endothelial cells cultured in the presence of serum from SLE and RA patients before and after RTX treatment. This effect, probably related to the re‐establishment of a homeostatic equilibrium in the immune system, and consequently on the vascular wall, might further contribute to prevent CVD in these autoimmune disorders.

A set of serum miRNAs, acting as regulators of potential key targets involved in the physiopathology of both disorders, were significantly reversed after RTX therapy. Moreover, that reversion paralleled the improvement of disease activity, as well as the changes occurred in the immune and inflammatory profiles, as well as the down‐regulation in the expression of NETs extrusion products.

The miRNAs validated by RT‐PCR in the whole cohort of RA patients have been previously reported to act as relevant regulators of immune cell development, playing a crucial role in the inflammatory response, and acting as key players in the pathogenesis of chronic and autoimmune disorders, including RA.^33^, ^34^

Consistent with our results, a previous study by our team found that after a 6‐month anti‐TNF‐α/DMARDs combination therapy, similar changes in three of the miRNAs found in the present RA cohort significantly increased in response to RTX (miR16‐5p, miR125a‐5p, and miR23a‐3p).^13^ The up‐regulation of those miRNAs was also associated with the improvement in the disease activity and the reduction in the levels of acute‐phase reactants after therapy.

On the other hand, this is the first study that identifies the changes promoted in the serum miRNA profile of SLE patients after treatment with RTX. The selected miRNAs were validated as up‐regulated after RTX treatment included main regulators of inflammation, atherosclerosis, CV disease and nephropathy (ie miR‐28‐5p, miR‐106‐3p and miR‐148b‐3p),^35^, ^36^, ^37^, ^38^ as well as inhibitors of IL‐6 production (ie miR‐151a‐3p).^39^ Similar in the case of RA, the changes promoted by RTX therapy in that miRNAs were found parallel to the changes promoted in both disease activity and the inflammatory profile of these patients. Overall, our data suggest that differentially expressed miRNAs in the serum of SLE and RA patients before and after 3 months of RTX therapy have potential to serve as novel biomarkers for monitoring therapy responsiveness. Intriguingly, RTX was able to induce an inflammatory status re‐assessment after only 3 months since induction therapy. One might speculate that changes in miRNAs profile might represent an early biomarker of clinical response when other conventional parameters lack adequate accuracy. In the future, these observations might guide strategic therapeutic options, especially when assessing treatment failure and/or poor response in order to identify tailored strategies.

In our series of patients, clinical effectiveness of RTX was demonstrated in a real‐life setting, with rapid improvement in SLE and RA signs and symptoms, only after 12 weeks of therapy. Although it is well recognized that complete remission is reached only after 6‐12 months of treatment, and therefore it is not possible to conclude the precise timescale of improvement in the serological indices measured, this study allowed us to demonstrate, as previously reported,^40^, ^41^ that a significant reduction of disease activity in both autoimmune conditions is already visible only after 3 months of treatment. This study also allowed us to recognize a number of cellular and molecular mechanisms underlying this early response.

Because of the heterogeneity of clinical manifestations, add‐on therapies and applied protocols, we could not investigate the impact of different RTX regimens on immune and vascular phenotypes. In future studies, larger study populations and longer‐term time‐points may identify additional important patient‐centred outcome and molecular targets.

Taken together, our data demonstrated that:

- SLE and RA patients display altered inflammatory, netotic and epigenetic profiles, which are interconnected and further related to the specific autoimmune profile and the activity of each disease.
- B lymphocytes play key roles in shaping pathological immune responses of SLE and RA.
- Associated with clinical response, RTX induced early restoration of homeostasis in immune and vascular systems.

## CONFLICT OF INTEREST

The authors declare no competing interests.

## AUTHOR CONTRIBUTIONS

C. P‐S, I. C, N. B, AM. P.T, M. L‐T, A. I‐C, Y. J‐G and I. A‐R developed the in vivo assays, performed the experiments and solved technical problems. L. P‐S, R. O, A. E, MC. C, M. R, MJ. C, D. R, S. S and MA. A followed up with patients and contributed useful discussion and suggestions. C.P‐S, I. C, N. B, E.C and C.L‐P formed the hypothesis, directed and coordinated the project, designed the experiments, analysed the data and wrote the manuscript. E. C, Y.J‐G, M. R, S. S and I. C performed statistical analysis and discussed results.

## APPENDIX 1

### 1.1

The members of the BIOSAR study group include the following: Jose Luis Marenco, Julia Uceda, JosePerez‐Venegas, Mª Dolores Ruiz‐Montesinos, Carlos Rodriguez Escalera, Antonio Fernández‐Nebro, Natalia Mena‐Vazquez, Carmen Romero‐Barco, Jerusalem Calvo, Rocío Segura‐Ruiz and Pilar Font‐Ugalde.
