# Supplementary material for: Early restoration of immune and vascular phenotypes in systemic lupus erythematosus and rheumatoid arthritis patients after B cell depletion
Source: J Cell Mol Med. 2019 Jul 26;23(9):6308–18. doi: 10.1111/jcmm.14517 (PMC6714224; doi:10.1111/jcmm.14517)
Supplement: Supplementary file 1 [file JCMM-23-6308-s001.pptx]

## Slide 1
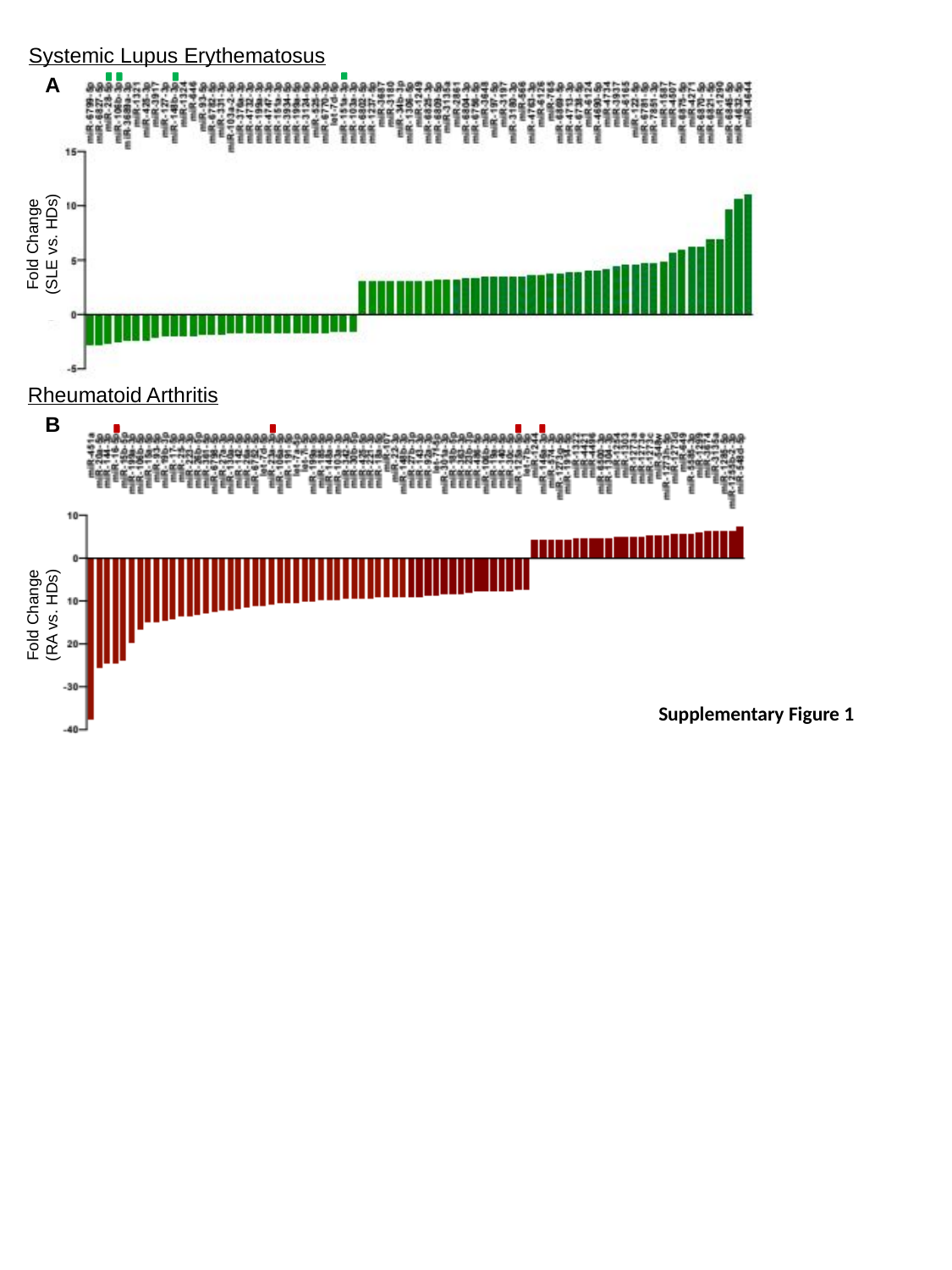

Systemic Lupus Erythematosus
A
Fold Change
(SLE vs. HDs)
Rheumatoid Arthritis
B
Fold Change
(RA vs. HDs)
Supplementary Figure 1

## Slide 2
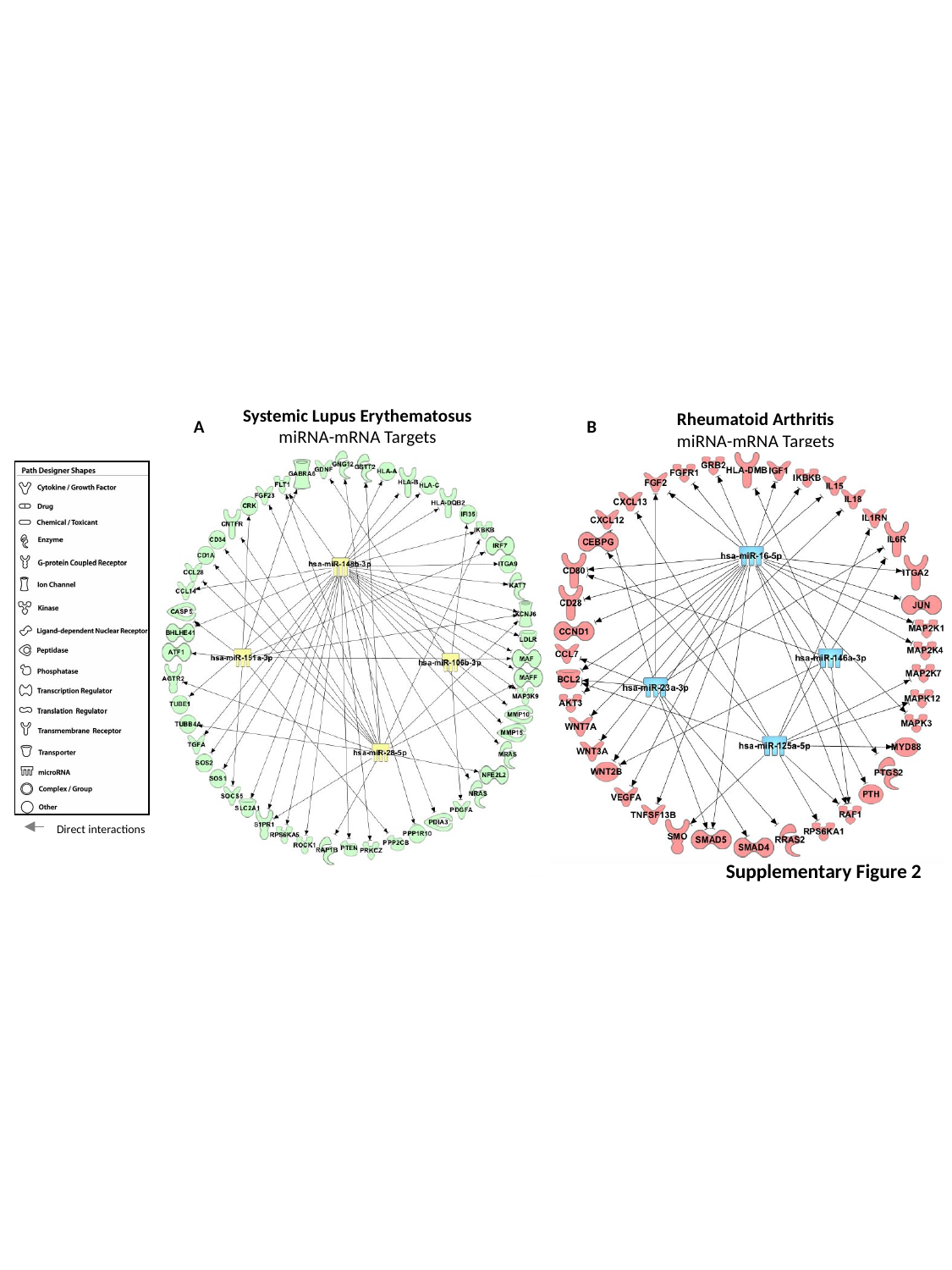

Systemic Lupus Erythematosus
miRNA-mRNA Targets
Rheumatoid Arthritis
miRNA-mRNA Targets
A
B
SLE
Direct interactions
Supplementary Figure 2

## Slide 3
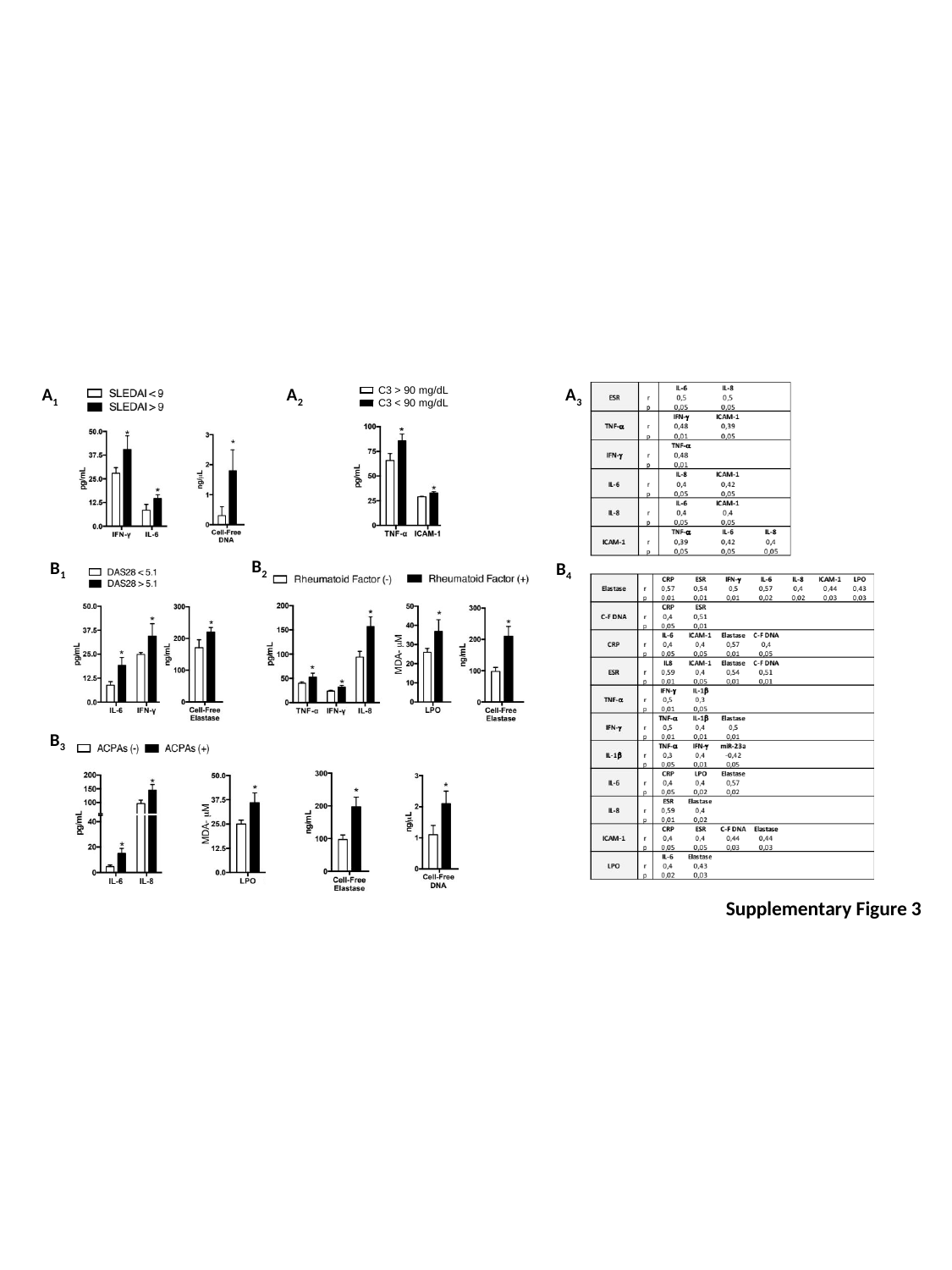

A1
A2
C3 > 90 mg/dL
A3
C3 < 90 mg/dL
B2
B1
B4
B3
Supplementary Figure 3

## Slide 4
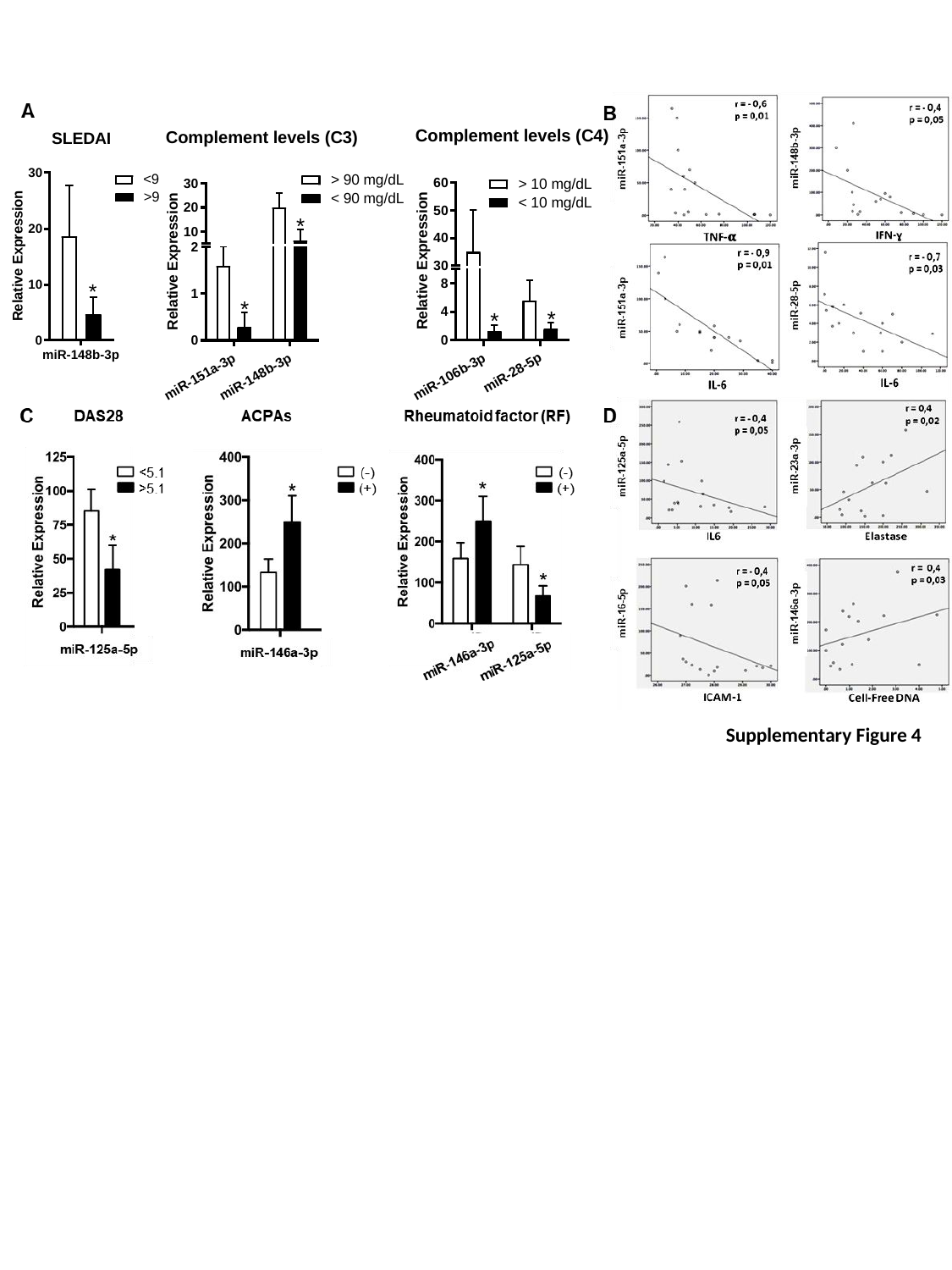

Supplementary Figure 4

## Slide 5
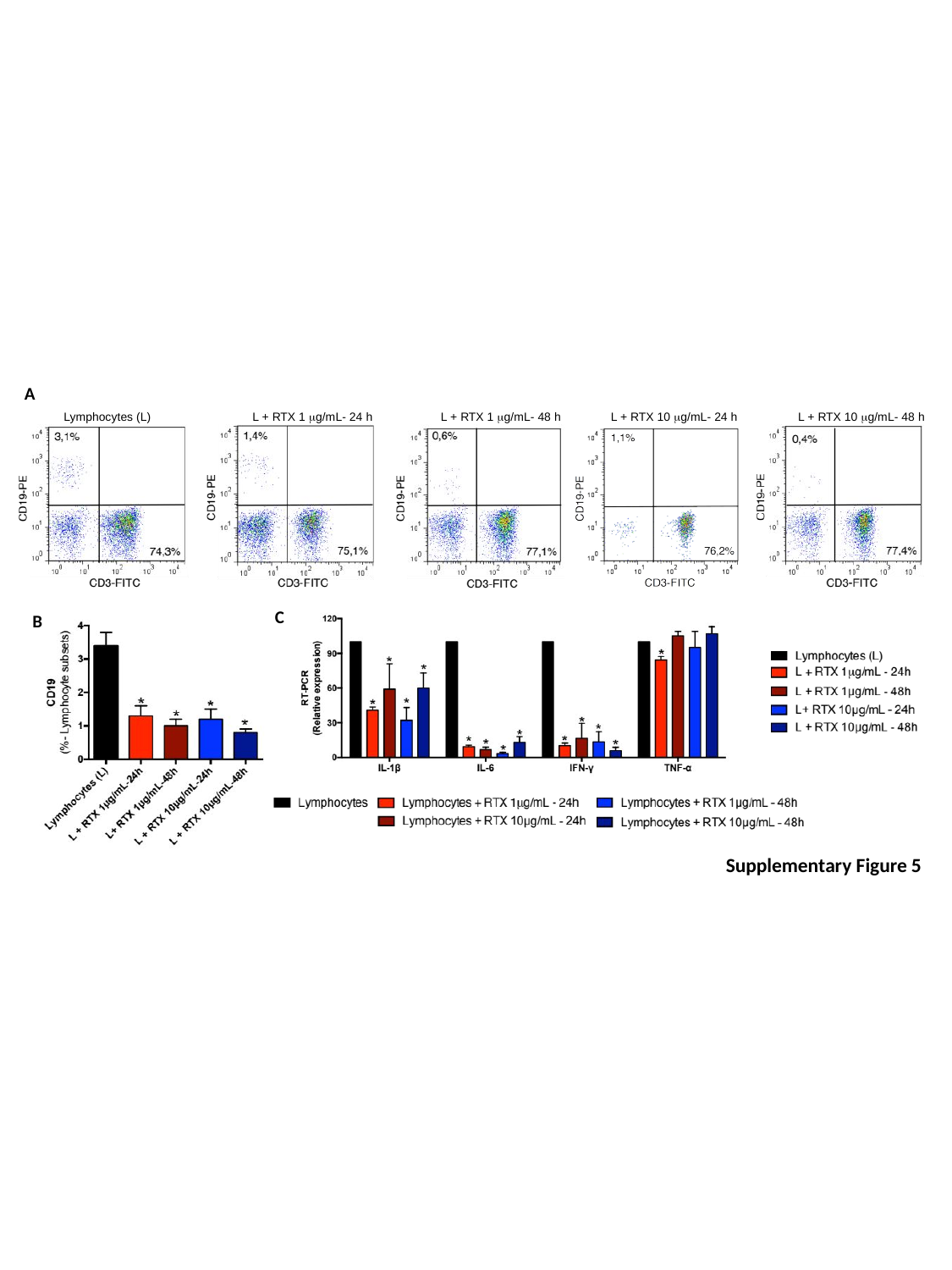

A
Lymphocytes (L)
L + RTX 1 mg/mL- 24 h
L + RTX 1 mg/mL- 48 h
L + RTX 10 mg/mL- 24 h
L + RTX 10 mg/mL- 48 h
C
B
Supplementary Figure 5

## Slide 6
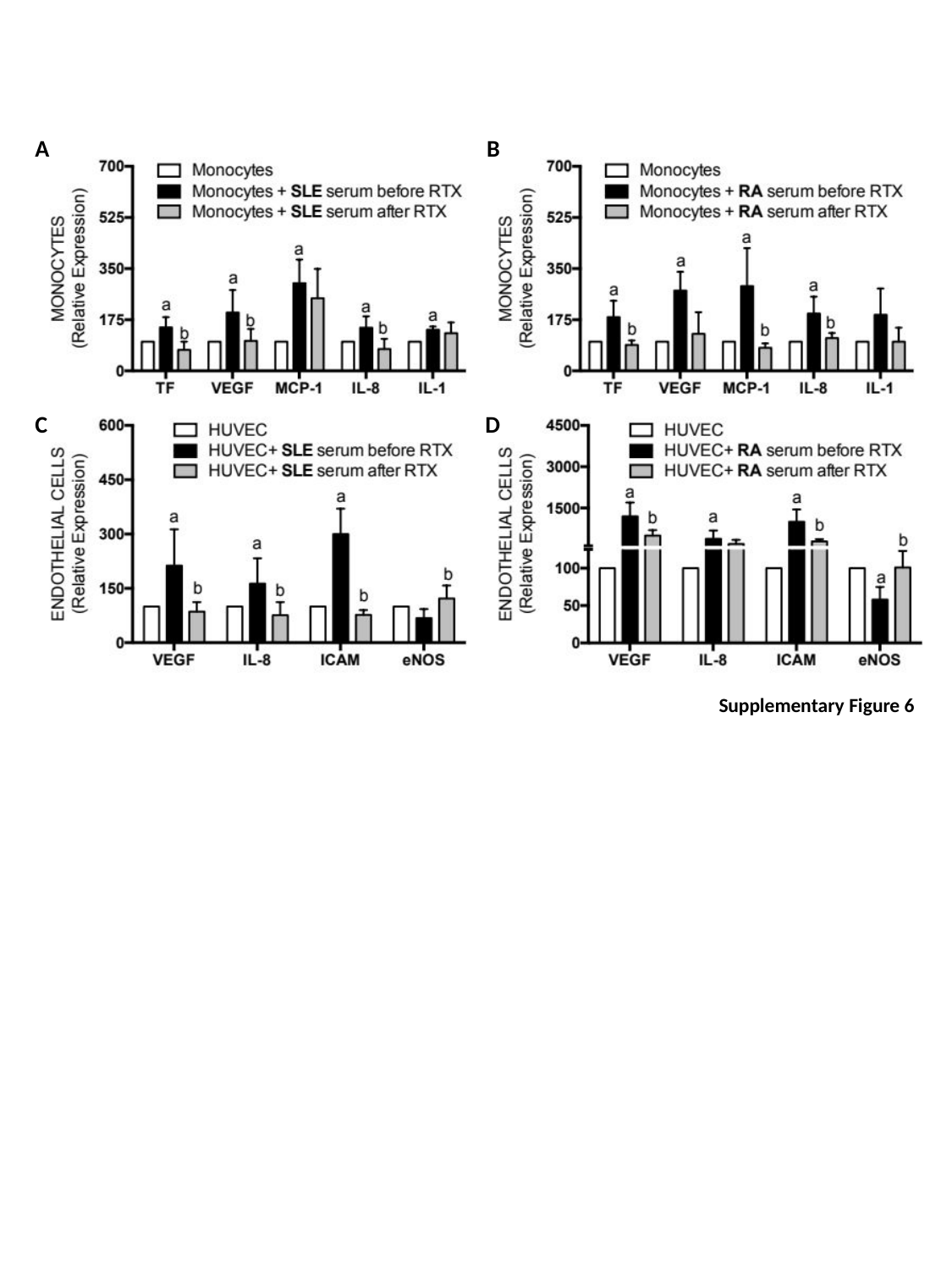

A
B
C
D
Supplementary Figure 6

## Slide 7
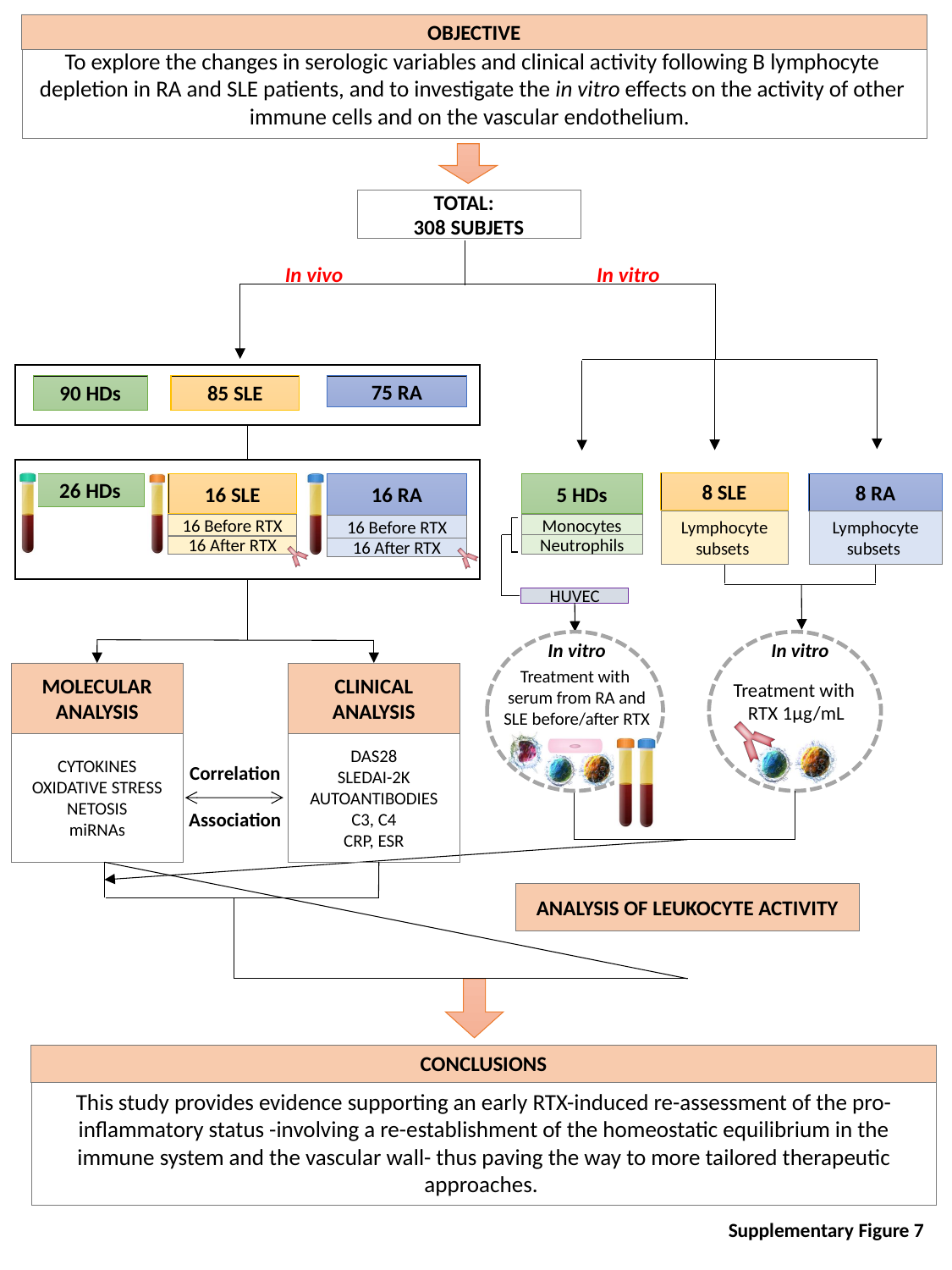

OBJECTIVE
To explore the changes in serologic variables and clinical activity following B lymphocyte depletion in RA and SLE patients, and to investigate the in vitro effects on the activity of other immune cells and on the vascular endothelium.
TOTAL:
308 SUBJETS
In vitro
In vivo
90 HDs
85 SLE
75 RA
8 SLE
Lymphocyte
subsets
26 HDs
16 SLE
16 Before RTX
16 After RTX
16 RA
16 Before RTX
16 After RTX
5 HDs
Monocytes
Neutrophils
8 RA
Lymphocyte
subsets
HUVEC
In vitro
Treatment with
serum from RA and
SLE before/after RTX
In vitro
Treatment with
RTX 1µg/mL
MOLECULAR ANALYSIS
CYTOKINES
OXIDATIVE STRESS
NETOSIS
miRNAs
CLINICAL ANALYSIS
DAS28
SLEDAI-2K
AUTOANTIBODIES
C3, C4
CRP, ESR
Correlation
Association
ANALYSIS OF LEUKOCYTE ACTIVITY
CONCLUSIONS
This study provides evidence supporting an early RTX-induced re-assessment of the pro-inflammatory status -involving a re-establishment of the homeostatic equilibrium in the immune system and the vascular wall- thus paving the way to more tailored therapeutic approaches.
Supplementary Figure 7
