# Supplementary material for: Early restoration of immune and vascular phenotypes in systemic lupus erythematosus and rheumatoid arthritis patients after B cell depletion
Source: J Cell Mol Med. 2019 Jul 26;23(9):6308–18. doi: 10.1111/jcmm.14517 (PMC6714224; doi:10.1111/jcmm.14517)
Supplement: Supplementary file 2 [file JCMM-23-6308-s002.docx]

**Supplementary Information**

**Early restoration of immune & vascular phenotypes in Systemic Lupus Erythematosus and Rheumatoid Arthritis patients after B-cell depletion**

Carlos Pérez-Sánchez^*^, Irene Cecchi^*^, Nuria Barbarroja, Alejandra M. Patiño-Trives, María Luque-Tévar, Laura Pérez-Sánchez, Alejandro Ibáñez-Costa, Iván Arias de la Rosa, Yolanda Jiménez-Gómez, Rafaela Ortega, Alejandro Escudero, Mª Carmen Castro, Massimo Radin, Mª José Cuadrado, Dario Roccatello, Savino Sciascia, Mª Ángeles Aguirre, Eduardo Collantes^#^, and Chary Lopez-Pedrera^#^.

**Supplementary Material and Methods**

**Blood collection and assessment of inflammatory and oxidative stress parameters**

Plasma and serum samples, and purified neutrophils, lymphocytes and monocytes for *in vitro* studies, were obtained from peripheral venous blood samples. To avoid blood composition changes promoted by diet and circadian rhythms, samples were always collected in the early hours in the morning and after a fasting period of 8 hours.

Interferon gamma (IFN-γ), Interleukins (IL) IL-1, IL-2, IL-6, IL-8, IL-10, IL-17, IL-23, monocyte chemotactic protein-1 (MCP-1), Macrophage Inflammatory protein 1a (MIP-1α), Tumor Necrosis Factor alpha (TNF-α), tissue plasminogen activator (tPA), Vascular Endothelial Grow Factor (VEGF) A, and soluble platelet selectin (sP-selectin) levels were quantified in sera using a cytofluorimetry-based ELISA system (Flowcytomix, Bender Medsystem GmbH, Austria). Two-colour cytometric analysis was performed using FACScalibur cytometer (Becton Dickinson Immunocytometry Systems (BDIS); San José, CA). Data were obtained and analysed using the FlowCytomix Pro software.

Assays of lipid peroxidation (lipoperoxides, LPO) levels were carried out by using thiobarbituric acid reactive substance (TBARS) Assay (Canvax Biotech, Córdoba, Spain) following manufacturer’s instructions.

Plasma total antioxidant capacity (TAC) was analyzed by quantitative colorimetric determination, using TAC Assay kit (BioVision, Mountain View, CA, USA).

**Measurement of NETs components in serum of SLE and RA patients before and after RTX treatment and NETs immunofluorescence staining and quantification**

Cell-free neutrophil elastase levels were measured in serum from patients and HDs or supernatants from *in vitro* studies, using the Human PMN Elastase ELISA Kit (Abcam, Cambridge, UK) following the manufacturer’s recommendations.

Cell-free DNA was evaluated by using SYTOX green in a fluorescence microplate-based assay. DNA concentrations were calculated based on a standard curve (0-5 ng/µL) of known concentrations of DNA (DNA from calf thymus; Sigma-Aldrich, St Louis, MO, USA) diluted in TBS.

For NETs quantification, neutrophils isolated from HDs incubated with serum from SLE or RA patients obtained at baseline and after 3 months of RTX treatment, were stained with neutrophil elastase (NE) (Abcam) and DAPI as nuclear staining (Invitrogen, UK), as previously described. Recorded images in a Nikon Eclipse-Ti-S fluorescent microscope were then analyzed using the IMAGE-J software. The number of cells positive for both, NE and DAPI were considered a NET and digitally recorded to prevent multiple counts. The percentage of NETs was calculated as the average of five to six fields (x40) normalized to the total number of cells. Results were expressed as percentage of NETs (NETs formation).

**Isolation of miRNAs from serum, miRNA expression profiling and quantitative real-time PCR.**

To identify the changes that occurred in the expression levels of miRNAs in serum from SLE and RA patients and HDs, an array was performed on an exploratory cohort -including 3 samples from clinically representative RA patients, 3 from SLE patients and 3 from HDs- using the HTG EdgeSeq miRNA whole transcriptome assay (miRNA WTA), which enabled to measure the expression of 2,083 human miRNA transcripts using next generation sequencing (NGS) (HTG Molecular technologies, Tucson, AZ, USA). To adjust for variations in RNA extraction and/or copurification of inhibitors, 5fmol of spike-in non-human synthetic miRNA (*C. elegans* miR-39 miRNA mimic: 5´- UCACCGGGUGUAAAUCAGCUUG-3´) were added to the samples after the initial denaturation.

The expression levels of serum miRNAs were validated by real time PCR (RT-PCR) using a LightCycler® Thermal Cycler System (Roche Diagnostics, Indianapolis, Indiana, USA). Specifically, 3µl of RNA eluate were reverse transcribed in 10µl reactions using the miRCURY LNATM Universal RT mi-RNA PCR, Polyadenylation and cDNA synthesis kit (Exiqon, Vedbaek, Denmark). RT-PCR was carried out with 4µL cDNA diluted 40x and 6µL of reaction mixture [5µL of SYBR Green master mix (Exiqon) and 1µL of the corresponding PCR primer mix (microRNAs LNATM PCR primer set, Exiqon)]. After an initial hold of 10 minutes at 94ºC, samples were cycled 40 times at 95º for 10 seconds and at 60ºC for 1 minute. The expression levels of miRNAs were normalized to the mean of spiked-in miRNA Cel-miR-39. The expression levels of miRNA were calculated using the 2-∆∆Ct method. All measurements were performed in duplicate. Controls consisting of reaction mixture without cDNA were negative in all runs. List of miRNA sequences is displayed in Supplementary Table 3.

**Supplementary Tables**

**Supplementary Table 1. Clinical details of healthy donors, and SLE and RA patients.** SLE, Systemic Lupus Erythematosus; RA, Rheumatoid Arthritis; ACPAs, Anti citrullinated protein antibodies; RF, Rheumatoid Factor; CRP, C Reactive Protein; DAS28, RA Disease Activity Score; SLEDAI-2K, Systemic Lupus Erythematosus Disease Activity Index; NSAIDS, non-steroid anti-inflammatory drugs. Immunosuppressors include methotrexate and leflunomide in RA patients, and mycophenolate, azathioprine and methotrexate in SLE patients. ***P**** indicates significant differences vs healthy donors (P<0.05).

|  | **Healthy**  **Donors**  (n=60) | **SLE**  **Patients**  (n=85) | ***P**** |  | **Healthy**  **Donors**  (n=30) | **RA**  **Patients**  (n=75) | ***P**** |
| --- | --- | --- | --- | --- | --- | --- | --- |
| **Demographic Characteristics** | | | | | | | |
| Female/male (n) | 42/18 | 76/9 |  |  | 21/9 | 53/22 |  |
| Disease evolution, years  (mean±SD) |  | 9.1 ± 8.1 |  |  |  | 12.3 ± 8.9 |  |
| Age, years (mean±SD) | 38 ± 10 | 40 ± 12 |  |  | 46 ± 8 | 55 ± 11 |  |
| **Clinical Characteristics** | | | | | | | |
| Smoking (n, %) | 10 (16.6%) | 29 (34%) | **0.01** |  | 3 (10%) | 17 (22.6%) | **0.00** |
| Hyperlipidemia (n, %) | 2 (3.3%) | 19 (22.3%) | **0.01** |  | 1 (3.3%) | 37 (49.3%) | **0.00** |
| Arterial hypertension (n, %) | 1 (1.6%) | 14 (16,4%) | **0.01** |  | 2 (6.6%) | 21 (28%) | **0.00** |
| Nephropathy (n, %) | 0/60 (0%) | 25/85 (29%) | **0.00** |  |  |  |  |
| **Autoimmune profile** | | | | | | | |
| ACPAs, IU/mL (mean±SD) |  |  |  |  | 4.1 ± 3 | 61.4 ± 105.2 | **0.01** |
| RF, IU/mL (mean±SD) |  |  |  |  | 2.9 ± 2.1 | 147.1 ± 230 | **0.00** |
| Anti-dsDNA (n, %) | 0 (0%) | 20 (23.5%) | **0.00** |  |  |  |  |
| Antiphospholipid antibodies (n, %) | 0 (0%) | 24 (28.2%) | **0.00** |  |  |  |  |
| **Laboratory parameters** | | | | | | | |
| CRP (mg/L) (mean±SD) | 1.2 ± 1.2 | 3.6 ± 6.3 | **0.01** |  | 1.15 ± 1.4 | 14.3 ± 22.5 | **0.00** |
| C3 (mg/dL) (mean±SD) | 141.6 ± 45.6 | 114.8 ± 37.25 | **0.02** |  |  |  |  |
| C4 (mg/dL) (mean±SD) | 28.15 ± 11.8 | 18.8 ± 8.1 | **0.01** |  |  |  |  |
| **Disease Activity Assessment** | | | | | | | |
| DAS28 (mean±SD) |  |  |  |  |  | 3.4 ± 1.5 |  |
| SLEDAI-2K (mean±SD) |  | 2.3 ± 2.9 |  |  |  |  |  |
| **Treatments** | | | | | | | |
| NSAIDs (%) |  |  |  |  |  | 59 (98.3%) |  |
| Corticosteroids (n, %) |  | 57 (67.1%) |  |  |  | 41 (68.3%) |  |
| Immunosuppressors (n, %) |  | 59 (69.4%) |  |  |  | 53 (88.3%) |  |
| Anticoagulants/Antiplatelets agents (n, %) |  | 17 (20%) |  |  |  |  |  |

**Supplementary Table 2.** List of Primers

| **Gene** | **Sequence** | |
| --- | --- | --- |
|  | **Forward** | **Reverse** |
| GAPDH | TGTAGTTGAGGTCAATGAAGGG | ACATCGCTCAGACACCATG |
| IL6 | GCCCCACACAGACAGCCACTCACC | TGCCTCTTTGCTGCTTTCACACAT |
| IFNG | GGTAACTGACTTGAATGTCC | TTTTCGCTTCCCTGTTTAG |
| TNF | TCAGCTTGAGGGTTTGCTAC | TGCACTTTGGAGTGATCGG |
| IL8 | GGTGCAGAGGGTTGTGGAGAAGTT | CATGAAGTGTTGAAGTAGATTTGC |
| TF | TACTGTTTCAGTGTTCAAGCAGTGA | CAGTGCAATATAGCATTTGCAGTAGC |
| ICAM1 | GGGGAACCAGAGCCAGGAGACACT | TGGGCCTCACACTTCACTGTCACC |
| eNOS | CTCATGGGCACGGTGATG | ACCACGTCATACTCATCCATACAC |
| VEGFA | CGAAGTGGTGAAGTTCATGGATG | TTCTGTATCAGTCTTTCCTGGTGA |

**Supplementary Table 3.** List of miRNA sequences

| **miRNA** | **Sequence** |
| --- | --- |
| miR-151a-3p | CUAGACUGAAGCUCCUUGAGG |
| miR-148b-3p | UCAGUGCAUCACAGAACUUUGU |
| miR-106b-3p | CCGCACUGUGGGUACUUGCUGC |
| miR-28-5p | AAGGAGCUCACAGUCUAUUGAG |
| miR-146a-3p | CCUCUGAAAUUCAGUUCUUCAG |
| miR-125a-5p | UCCCUGAGACCCUUUAACCUGUGA |
| miR-16-5p | UAGCAGCACGUAAAUAUUGGCG |
| miR-23a-3p | AUCACAUUGCCAGGGAUUUCC |

**Supplementary Table 4. Correlations among miRNAs and inflammation and NETosis-derived products, as well as with clinical features of the disease in SLE and RA patients after RTX treatment.** IFN-γ, Interferon gamma; ESR, erythrocyte sedimentation rate IL, Interleukin; TNF-α, Tumor Necrosis Factor alpha; SLEDAI-2K, Systemic Lupus Erythematosus Disease Activity Index; RF, Rheumatoid Factor.

| **Systemic Lupus Erythematosus** | | | | | | | |
| --- | --- | --- | --- | --- | --- | --- | --- |
| **miR-151a-3p** |  | **IFN-γ** | **miR148b-3p** | **miR28-5p** |  |  |  |
|  | r | -0.695 | 0.638 | 0.587 |  |  |  |
|  | p | 0.043 | 0.044 | 0.047 |  |  |  |
| **miR-148b-3p** |  | **IFN-γ** | **Elastase** | **Cell-free DNA** | **SLEDAI-2K** | **miR151a-3p** | **miR28-5p** |
|  | r | -0.478 | -0.710 | -0.840 | -0.684 | 0.638 | 0.712 |
|  | p | 0.033 | 0.049 | 0.040 | 0.042 | 0.044 | 0.021 |
| **miR-106b-3p** |  | **C3** | **Elastase** |  |  |  |  |
|  | r | -0.681 | -0.841 |  |  |  |  |
|  | p | 0.042 | 0.049 |  |  |  |  |
| **miR-28-5p** |  | **SLEDAI-2K** | **miR151a-3p** | **miR148b-3p** |  |  |  |
|  | r | -0.479 | 0.587 | 0.712 |  |  |  |
|  | p | 0.032 | 0.047 | 0.021 |  |  |  |
|  | | | | | | | |
| **Rheumatoid Arthritis** | | | | | | | |
| **miR-146a-3p** |  | **TNF-α** | **ESR** | **RF** |  |  |  |
|  | r | 0.976 | 0.800 | 0.800 |  |  |  |
|  | p | 0.024 | 0.049 | 0.047 |  |  |  |
| **miR-125a-5p** |  | **IFN-γ** | **Elastase** | **miR-23a-3p** |  |  |  |
|  | r | -0.829 | -0.842 | 0.886 |  |  |  |
|  | p | 0.042 | 0.036 | 0.019 |  |  |  |
| **miR-16-5p** |  | **TNF-α** | **IFN-γ** | **IL-6** | **RF** |  |  |
|  | r | -0.791 | -0.900 | -0.697 | -0.900 |  |  |
|  | p | 0.049 | 0.037 | 0.041 | 0.037 |  |  |
| **miR-23a-3p** |  | **IFN-γ** | **IL-8** | **miR-16-5p** | **miR-125a-5p** |  |  |
|  | r | -0.886 | -0.667 | 0.904 | 0.886 |  |  |
|  | p | 0.019 | 0.039 | 0.035 | 0.019 |  |  |

**Supplementary Figure Legends**

**Supplementary Figure 1**. **Circulating microRNAs in SLE and RA patients after RTX therapy.** HTG EdgeSeq miRNA whole transcriptome assay was performed in serum samples from HDs, SLE and RA patients. Differentially expressed circulating miRNAs in SLE (A) and RA (B) patients showing fold change>2.

**Supplementary Figure 2.** **Interaction Network of microRNAs identified, and potential mRNA targets involved in clinical features of SLE and RA.** By using the tool microRNA Target Filter of QIAGEN’s Ingenuity Pathway Analysis (IPA, QIAGEN Redwood City, www.qiagen.com/ingenuity), the software generated a network including the selected microRNAs (miRNAs or miR) and their mRNA targets involved in the physiopathology of SLE (A) and RA (B). Only targets experimentally observed and predicted with high confidence are shown and related by direct interactions with their specific potential miRNA regulators.

**Supplementary Figure 3. Association and correlation studies in SLE and RA patients treated with RTX.** (A) Association studies of the altered serum markers related to inflammation, oxidative stress and NETosis, with SLE disease activity (based on the median of SLEDAI-2K value from the SLE cohort) (A1) and with the presence of hypocomplementemia C3 (A2). Bar graphs represent the mean ± Standard Deviation. (*) indicates significant differences at *P*<0.05. (A3) Correlation studies among the inflammatory parameters analyzed in the SLE serum. (r) indicates Spearman rank correlation coefficient; (p) indicates *P-*values.

(B1) Association studies of the altered serum markers related to inflammation, oxidative stress and NETosis, with RA disease activity (high activity defined by DAS28 > 5.1 based on EULAR criteria) and with the presence of RF (B2) and ACPAs (B3) positivity. Bar graphs represent the mean ± Standard Deviation. (*) indicates significant differences at *P*<0.05. (B4) Correlation studies between the inflammatory, oxidative stress and Netosis-derived biomolecules analyzed in the RA serum. (r) indicates Spearman rank correlation coefficient; (p) indicates *P-*values.

**Supplementary Figure 4. Association and correlation studies of miRNAs validated in SLE and RA patients.** Association studies of the altered miRNAs validated in the serum of SLE patients with SLEDAI-2K, hypocomplementemia C3 and C4 (A). Bar graphs represent mean±SD. (* *P*<0.05). (B) Correlation studies among the validated miRNAs and inflammatory parameters deregulated in SLE patients. (C) Association studies of the altered miRNAs validated in the serum of RA patients with DAS28, positivity for anti-citrullinated protein antibodies (ACPAs), and positivity for rheumatoid factor. Bar graphs represent mean±SD. (* *P*<0.05). (D) Correlation studies among the miRNAs validated and inflammatory and NETosis-derived parameters deregulated in RA patients. In B and D (r) indicates Spearman rank correlation coefficient; (p) indicates *P-*values.

**Supplementary Figure 5**. **Dose- and time-response experiments of RTX on RA-purified Lymphocytes.** Lymphocyte population was purified from RA patients and cultured with two doses of RTX (1 and 10 μ/mL) for two periods of time (24 and 48 hours) to evaluate the effect of B-cell depletion on the activity of other immune cells participating in the inflammatory response. (A) Flow cytometry dot-plot showing the staining with PE anti-CD19 and FITC anti-CD3 antibodies. (B) B-cell depletion analysis through the different doses and times of RTX-*in vitro* treatment. (C) Inflammatory modulation of lymphocyte population after RTX treatment by RT-PCR. Bar graphs represent the mean ± Standard Deviation. (*) indicates significant differences at *P*<0.05.

**Supplementary Figure 6. *In vitro* effects of RTX on the activation status of endothelial cells and monocytes by SLE and RA serum.** Monocytes isolated from healthy donors and Human Umbilical Vein Endothelial Cells (HUVEC) were stimulated with 10% of SLE (A-B) and RA serum before RTX therapy (C-D). The treatment with serum after RTX-therapy significantly reduced the expression of a panel of monocytes and HUVEC-activation markers by RT-PCR. Bar graphs represent the mean±SD. (a) indicates significant differences vs healthy donors (P<0.05). (b) indicates significant differences vs samples before RTX (P<0.05).

**Supplementary Figure 7. Flow chart of the study.** The total number of blood sample analyzed in the study was 111. A panel of markers related to inflammation, oxidative stress, miRNA and netosis was evaluated in the *in vivo* study in 90 serum samples from 25 HDs, 16 SLE patients and 16 RA patients before and after RTX therapy. Correlation and association studies were performed with clinical parameters. In addition, *in vitro* studies were carried out in cell samples from 5 HDs, 8 SLE patients and 8 RA patients. Lymphocytes population was treated in vitro with RTX. Monocyte, neutrophils and endothelial cells were treated with serum from SLE and RA patients before and after three months of RTX therapy. The effects of RTX on the leukocytes activation status was analyzed. HDs, Healthy Donors; SLE, Systemic Lupus Erythematosus; RA, Rheumatoid Arthritis; RTX, Rituximab; HUVECs Human Umbilical Vein Endothelial Cells; DAS28, RA Disease Activity Score; SLEDAI, Systemic Lupus Erythematosus Disease Activity Index; CRP, C Reactive Protein; ESR, Erythrocyte sedimentation rate; miR, microRNA.
